# Visual cues, expectations, and sensorimotor memories in the prediction and perception of object dynamics during manipulation

**DOI:** 10.1007/s00221-019-05711-y

**Published:** 2020-01-13

**Authors:** Thomas Rudolf Schneider, Gavin Buckingham, Joachim Hermsdörfer

**Affiliations:** 1 Chair of Human Movement Science, Department of Sport and Health Sciences, Technical University of Munich, Georg-Brauchle-Ring 60/ 62, 80992 Munich, Germany; 2 Sport and Health Sciences, College of Life and Environmental Sciences, University of Exeter, Heavitree Road, Exeter, EX1 2LU UK

**Keywords:** Grasping, Visual processing, Sensorimotor memories, Torque perception, Weight perception

## Abstract

When we grasp and lift novel objects, we rely on visual cues and sensorimotor memories to predictively scale our finger forces and exert compensatory torques according to object properties. Recently, it was shown that object appearance, previous force scaling errors, and previous torque compensation errors strongly impact our percept. However, the influence of visual geometric cues on the perception of object torques and weights in a grasp to lift task is poorly understood. Moreover, little is known about how visual cues, prior expectations, sensory feedback, and sensorimotor memories are integrated for anticipatory torque control and object perception. Here, 12 young and 12 elderly participants repeatedly grasped and lifted an object while trying to prevent object tilt. Before each trial, we randomly repositioned both the object handle, providing a geometric cue on the upcoming torque, as well as a hidden weight, adding an unforeseeable torque variation. Before lifting, subjects indicated their torque expectations, as well as reporting their experience of torque and weight after each lift. Mixed-effect multiple regression models showed that visual shape cues governed anticipatory torque compensation, whereas sensorimotor memories played less of a role. In contrast, the external torque and committed compensation errors at lift-off mainly determined how object torques and weight were perceived. The modest effect of handle position differed for torque and weight perception. Explicit torque expectations were also correlated with anticipatory torque compensation and torque perception. Our main findings generalized across both age groups. Our results suggest distinct weighting of inputs for action and perception according to reliability.

**Electronic supplementary material:**

The online version of this article (10.1007/s00221-019-05711-y) contains supplementary material, which is available to authorized users.

## Introduction

To dexterously grasp and lift objects, it is important to plan the grasp execution according to object properties before sensory feedback about these become available. While sensorimotor memories of previous lifts of well-known objects can be retrieved by object identification (Hermsdörfer et al. 2011), humans rely on visual cues like size (Cole 2008; Gordon et al. 1991) and material (Buckingham et al. 2009) to predictively scale the fingertip forces according to the presumed weight of novel objects. When lifting objects with an asymmetric center of mass, it is necessary to exert compensatory torques at the moment of lift-off to prevent the object from tilting. It has been shown that subjects can successfully utilize the shape of objects to anticipate its torque (Fu and Santello 2012; Lee-Miller et al. 2016; Salimi et al. 2003). However, as previous studies only investigated two opposing object shapes, the rapid formation of memory links between shapes and torques might have confounded the evaluation of the contribution of visual processing in sensorimotor control mechanisms and the reliance on sensorimotor memories after the first lift (Fu and Santello 2015). Furthermore, it is not known how the addition of random torque variation interacts with the utilization of visual cues. The combination of a fixed visual cue about object dynamics with an additional unpredictable variation occurs frequently in real-life situations. For example, the position and orientation of the handle of a tea cup signals the torque direction, however the exact torque magnitude can be uncertain if the level of liquid is not visible.

Besides guiding motor planning, the object appearance also alters how we perceive the weight of hand-held objects. In the ‘size–weight’ illusion, subjects who lifted equally heavy cubes of different sizes persistently judged the smaller object to be heavier (Ellis and Lederman 1993; Flanagan and Beltzner 2000). Similarly, when lifting equally heavy objects with differing surface materials, subjects judge the object apparently made of a less dense material to be the heaviest (Buckingham et al. 2009). However, when judging well-known everyday objects of similar natural weights, subjects judged bigger objects to be heavier than smaller ones, leading to the assumption that explicit expectations are added and not contrasted with sensory feedback (Buckingham and MacDonald 2016). Regarding the perception of the mass distribution or torques, subjects were shown to be capable of correctly estimating the center of mass (CoM) of an assembled object based on the visible object configuration after hefting the object components (Craje et al. 2013; Lee-Miller et al. 2016), while viewing a mirrored image of an unbalanced hand-held object compromised the correct judgement of the direction of the CoM (Xu et al. 2012).

Apart from visual cues, implicit sensorimotor memories of object properties and the motor execution of prior lifts also affect anticipation as well as perception of object properties. The effect of sensorimotor memories has been demonstrated on anticipatory force scaling (Johansson and Westling 1988; Quaney et al. 2003) and torque compensation (Lukos et al. 2013) both in the absence and presence of explicit cues (Flanagan et al. 2008; Fu and Santello 2012). Excessive grip-force scaling based on sensorimotor memories is associated with lower heaviness ratings (van Polanen and Davare 2015). Recently, we found that torque planning errors biased the perception of both torques and weight and also inhibited the adaptation of subsequent anticipatory torque compensation when the CoM was unforeseeably varied in the absence of cues (Schneider et al. 2019). It remains to be investigated, however, how important planning errors are when relevant visual cues are present. Furthermore, very few studies have simultaneously evaluated the unique contributions of sensory and visual information, sensorimotor memories as well as explicit percepts and expectations to anticipatory grasp planning and object perception.

Elderly subjects were repeatedly shown to exert higher grip forces than young adults, and showed deficits in the adaptation of grip forces to changing surface properties (Cole 1991; Cole et al. 1999; Kinoshita and Francis 1996). The findings were partly explained by changes of skin properties leading to reduced finger-surface friction, but were also attributed to impaired cutaneous afferent encoding of skin–object frictional properties (Cole et al. 1999) as aging reduces sensory sensitivity (Konczak et al. 2012). Similarly, in a force-matching task, the sensory attenuation of self-generated forces increased with age—a finding the authors interpreted as an increased reliance on sensorimotor predictions as sensory precision decays (Wolpe et al. 2016). Concerning the processing of visual cues, old adults failed to learn to utilize color cues about object weight and surface friction to guide efficient fingertip forces (Cole and Rotella 2002). In contrast, in size- and material-weight illusion tasks, older and younger adults did not differ in the magnitude of their initial perceptual size- and material-weight illusions (Trewartha and Flanagan 2016). While these findings on the impact of aging on the processing of weight-related visual information remain inconclusive, the utilization of visible shape cues for the prediction of torques in motor planning and perception has not yet been investigated in the elderly.

Here, we had participants repeatedly grasp the handle of a compound object with a precision grip to lift it. Across trials, the CoM was altered by randomly positioning the handle across the horizontal base, while additionally randomly placing a hidden weight in the cavities of the base. Subjects had to prevent the object from tilting during the lift and to indicate the object’s CoM before and after lifting as well as giving a heaviness estimate after lifting it. By conducting multilevel, multivariable regression analyses, we analyzed the distinct contributions of visual shape cues, previously experienced torques, prior expectations, and committed torque planning errors to anticipatory torque compensation and the perception of torques and weight. To assess the impact of aging on the contribution of visual shape cues and sensorimotor memories in action and perception and to improve the generalizability of findings (Henrich et al. 2010), 12 young as well as 12 elderly subjects participated in the study.

Based on the results of previous studies, we hypothesized that geometric cues as well as sensorimotor memories of torques and torque planning errors will all correlate with subsequent torque compensation, whereas previous torque percepts and explicit expectations will not (Craje et al. 2013; Schneider et al. 2019). Regarding perception, we expected to replicate our previous findings in the absence of handle position changes that torque planning errors influence the perception of torques to a similar extent as the actual torque and cause an illusionary percept of heaviness (Schneider et al. 2019). In analogy to size–weight illusion studies, we predicted that the object shape may give rise to implicit predictive torque expectations which will be subtracted from the overall torque percept and also decrease the torque and error induced heaviness illusion. As the importance of predictive mechanisms was previously shown to increase with age as sensory precision fades (Wolpe et al. 2016), we presumed that elderly subjects would be more reliant on visual cues, with lesser dependence on sensed torques and sensorimotor memories.

## Materials and methods

### Participants

24 participants, consisting of 12 young (7 female, 9 right-handed, 18–26 years, mean age 22.8 ± 2.5 years) and 12 elderly (5 female, all right-handed, 62–76 years, mean age 68.4 ± 4.8 years) individuals with normal or corrected to normal vision took part in the experiment. Handedness was assessed by self-report. All subjects were naïve to the purpose of the study and gave informed consent to participate in the experiment. The experimental procedures were approved by the Institutional Review Board of the School of Medicine at the Technical University of Munich and were in accordance with the Declaration of Helsinki. Upon questioning, all the subjects did not report a history of neurological disorders or musculoskeletal disorders of the involved upper limb, nor did they report the intake of drugs which were classified as centrally acting by the experimenter. The participants received 20 € for their participation in the experiment which lasted for  ~ 2 h.

### Experimental design and statistical analyses

#### Experimental apparatus and procedure

**Fig. 1:**
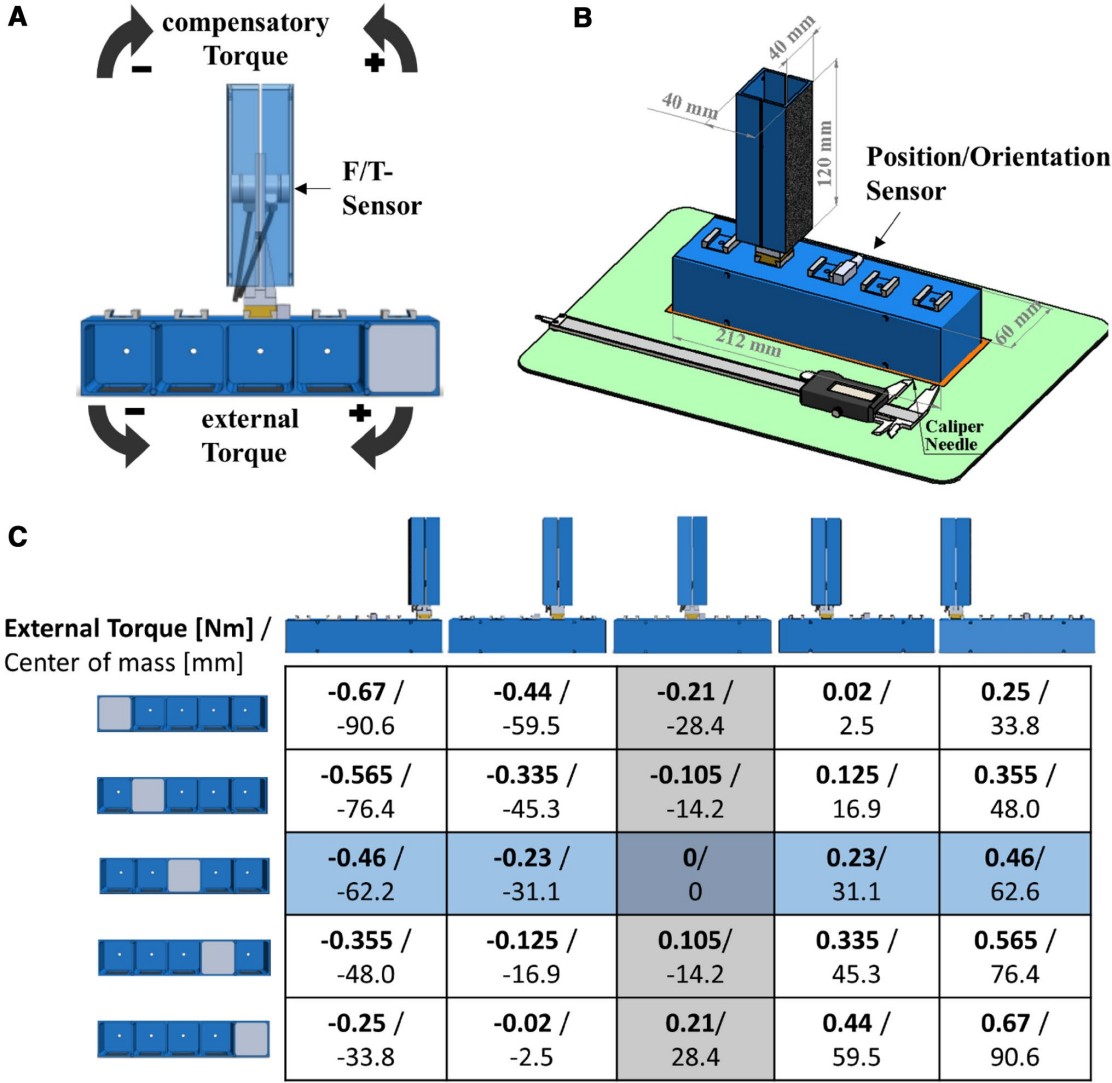
Experimental setup. The custom-built grip-device consists of a handle element which was randomly positioned on one of five slots along a horizontal bar via dove-tail linkups (**a**, **b**). The handle element allowed subjects to freely choose digit placement on the sandpaper (Bosch, P320) covered gasp surfaces (40 × 120 mm) (**b**). Additionally, a 250 g aluminum weight was randomly placed into the five cavities of the horizontal bar. The resulting horizontal centers of mass and external torques after lift onset for a vertical object orientation are denoted in **c** for each combination of handle and weight position. A detachable lid blocked the cavities from view (**b**). 6-axis force/torque sensors were mounted below the aluminum panels underneath the grasp surfaces, blocked from view (the panels are rendered transparent for illustrative purposes in **a**. A magnetic position/ orientation tracker was mounted centrally on the horizontal bar (**a**, **b**). To indicate their perception of the center of mass/the external torque, subjects used the needle of a digital caliper which was parallel to the horizontal bar and aligned with the right edge of the grip device (**b**)

A custom-made grip device consisted of a handle element with grasp surfaces (length 120 mm, width 40 mm) covered with fine grain sandpaper (Bosch, P320) allowing for free choice of digit placement. The handle could be flexibly mounted onto one of the five slots along the horizontal axis of a bar via dove-tail links. This bar (length 212 mm, height 40 mm, depth: 60 mm) contained five cavities (length and width 40 mm, depth 60 mm) which could be covered by a detachable aluminum lid. Two six-axis force/torque sensors were fitted underneath the grasp surfaces and concealed from sight by aluminum panels. A lightweight magnetic position/orientation tracker was fixed centrally on top of the horizontal bar (see Fig. 1a, b).

Prior to each trial, we asked the participants to close their eyes while we randomly mounted the handle element onto one of the five slots of the horizontal bar and randomly placed a 250 g aluminum weight, concealed by the detachable aluminum lid, into one of five cavities along the horizontal axis of the object base. We conducted these manipulations behind a laptop screen, shielding the object from view. The total object weight was 750 g including the aluminum weight. We calculated the center of mass (CoM) along the horizontal axis relative to the center line between the grasp surfaces and the resulting external torques around a sagittal line through the center for each combination of the handle and weight position with SolidWorks 2014 (Dessault Systems) assuming an upright object orientation (see Fig. 1b). Negative signs denote a CoM to the left of the grip middle and a resulting counter-clockwise external torque and positive signs accordingly denote a CoM to the right of the grip middle with a resulting clockwise external torque. The arising external torques amounted to − 0.460 Nm, − 0.230 Nm, 0 Nm, 0.230 Nm, and 0.460 Nm for the outer left-, middle-left-, middle-, middle-right- and outer right handle position, respectively, when the weight was placed in the middle cavity (mean weight position). This part of the total torques will be denoted as ‘external torque induced by handle position’ and used as the variable representing the object shapes which resulted for the distinct handle positions. The respective CoMs of the object were − 62.2 mm, − 31.1 mm, 0 mm, 31.1 mm, and 62.2 mm. The additional torque added by the random placement of the hidden weight accounted for a further − 0.210 Nm, − 0.105 Nm, 0 Nm, 0.105 Nm, and 0.210 Nm, corresponding to a shift of the CoM of − 28.4 mm, − 14.2 mm, 0 mm, 14.2 mm, and 28.4 mm for the weight being placed in the outer left, middle left, center, middle right, and outer right cavity (see Fig. 1b). Hence, neither the overall nor the average external torques could be inferred directly by the handle position as subjects did not know the mass or the mass distribution of the object base. Throughout the manuscript, we will refer to the overall external torque simply as ‘external torque’ and we will refer to the torque component which can be attributed to the visible object shape as ‘external torque induced by handle position’.

At the beginning of the experiment, to raise the expectation that total object weight might vary from trial to trial, the experimenter initially showed participants a set of two aluminum and two plastic weights and stated that each and any combination of these could be randomly placed in the five concealed cavities of the horizontal bar before each lift. Subsequently, all weights were hidden from view and only one aluminum weight was used.

At the start of each trial, participants had to indicate their expectation of the object’s center of mass, which was introduced as the edge at which the object was balanced without tilting, with the needle of a digital caliper (see Fig. 1b). Following the first signal tone, participants had to reach for and grasp the handle surfaces with the fingertips of the thumb, index, and middle finger of their dominant hand and lift the object in a smooth, natural movement straight upwards with the task goal to minimize object tilt. After lifting the object to a height of ~ 10 cm, they were asked to hold it steady in the air until a second tone 4 s after the first signaled them to replace the object on the mousepad. Subjects could freely position their fingers on the grasp surfaces. After each lift, subjects had to indicate the perceived CoM in the same way as before and to give the numerical value that they felt best represented the heaviness of the object they had just lifted. No constraints were placed on this value or its range other than larger numbers representing heavier weights (i.e., absolute magnitude estimation) (Zwislocki and Goodman 1980).

The experiment consisted of 100 object lifts, such that each of the 25 distinct handle weight configurations was encountered 4 times. The sequence of the handle and weight positions was randomly and independently determined for each participant using the ‘datasample’ function in Matlab 2016a (MATLAB, RRID:SCR_001622) beforehand.

#### Data recording and processing

**Fig. 2:**
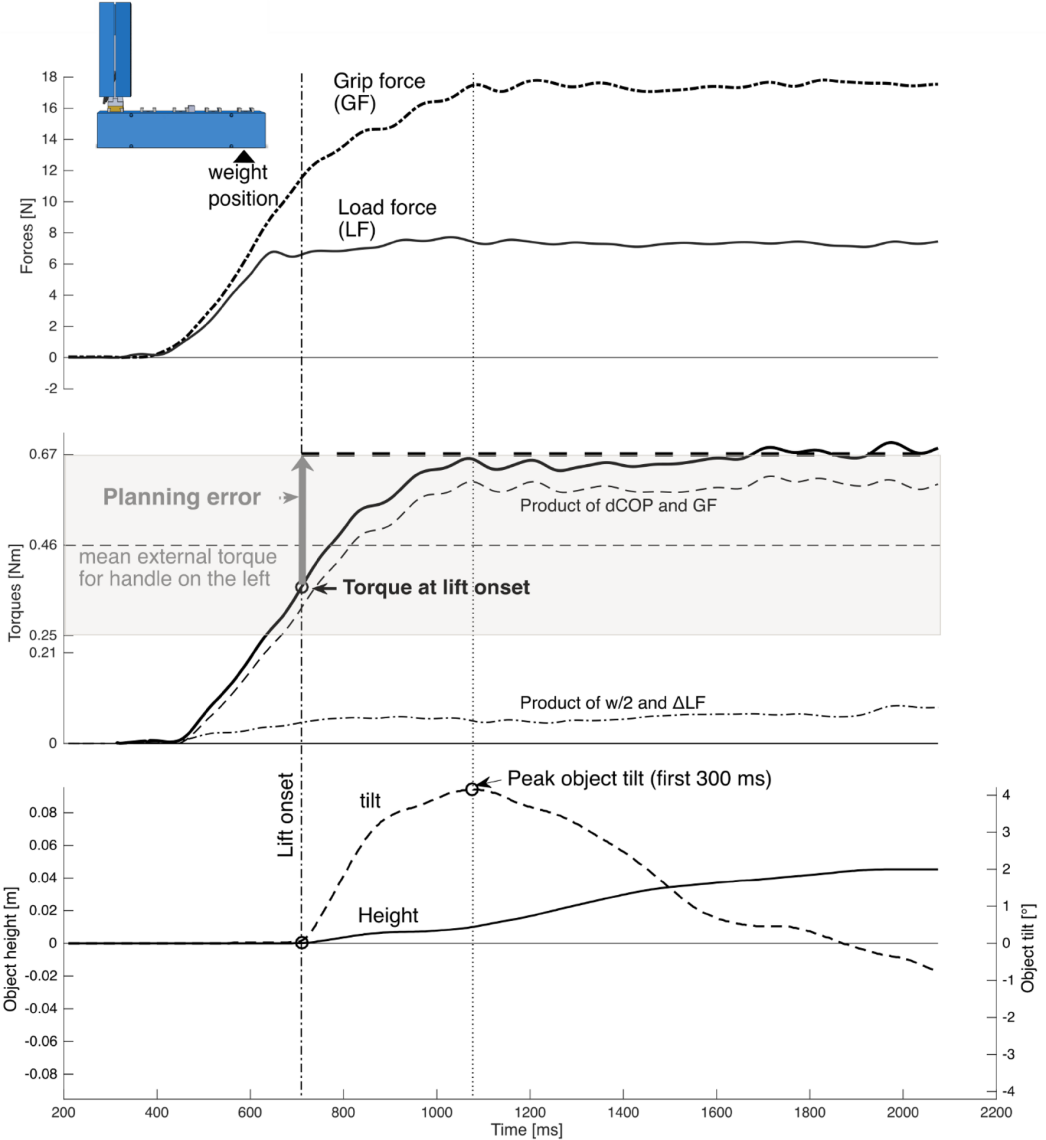
Representative trial illustrating the task variables. The upwards directed load force sum (LF) exceeds the gravitational force prior to object lift onset (vertical dash-dotted line). The mean grip force (GF) acting orthogonal towards the grasp surfaces causes friction preventing finger slip. To prevent object tilt, the total exerted torque must compensate for the external torque (horizontal dashed line in the torques subplot). The gray area shows the range of torque which may occur for the depicted handle position with the central horizontal line denoting the external torque for this handle position given a weight placement in the middle cavity. The planning error denotes the difference between the external torque and the exerted torque at lift onset, hence the uncompensated or net torque at lift onset. The planning error is highly correlated with the peak object tilt occurring in the first 300 ms after lift onset (vertical dashed line)

The signals of the two 6-axis force/torque sensors (ATI Nano-17 SI-50-0.5, ATI Industrial Automation; force range 50, 50, and 70 N for *x*-, *y*-, and *z*-axes, respectively; force resolution: 0.012 N; torque range 0.5 Nm; torque resolution: 0.063 Nm, sampling rate 200 Hz) which were digitally converted and transferred to a laptop by a Net-F/T-transducer box (ATI Industrial Automation) as well as the digital signal of the magnetic position and orientation sensor (TrakSTAR, Ascension Technology Corporation, accuracy: 1.4 mm RMS, 0.5 degrees RMS, sampling rate 200 Hz) were collected and synchronized using custom software written in Matlab 2012. Custom software written in Matlab 2016a was used for further data processing and variable extraction. The force/torque data were filtered through a zero-phase, sixth-order Butterworth low-pass filter with a cutoff frequency of 14 Hz. We calculated the compensatory torque which subjects exerted at the moment of object lift onset (*T*_com_) as an established indicator of motor prediction before full-sensory feedback about the object weight and weight distribution becomes available (Fu et al. 2010; Salimi et al. 2000). *T*_com_ is the sum of: (a) *T*_ΔLF*w/2_: the torque generated by the product of the difference between the right and left load force (the force directed upwards) and half the distance between the grasp surfaces ($\frac{w}{2}$ = 20 mm) and (b) *T*_ΔCoP*GF_: the product of the mean grip force (GF, the forces acting orthogonal to the grasp surfaces) and the difference between the right and left mean center of pressure of the grasping fingers on the grasp surfaces (ΔCoP). Following this convention *T*_com_ matches in sign with the external torque when it compensates for the exerted torque, e.g. is directed in opposing direction to the external torque. Hence, clockwise exerted torques were defined as negative and counter-clockwise torques as positive (see Fig. 1a). We refer to the Supplementary material of Schneider et al. 2019, https://doi.org/10.6084/m9.figshare.7683707, for details on the task mechanics and the calculations. The instant of object lift onset was determined as the moment 10 ms prior to which the vertical position of the object raised above a threshold of 0.2 mm, to minimize the detection lag after lift onset. The difference between the external torque and *T*_com_ is the uncompensated torque at lift onset and leads to an angular object acceleration after lift onset. We define this difference as torque planning error (see Fig. 2). The peak object tilt in the frontal plane in the first 300 ms after lift onset was extracted as performance measure of torque anticipation.

#### Data management

Due to technical errors (mostly delayed starts of recording and errors in the saving of the files) 1.75% (42/2400) of the measurements were faulty and the respective observations were discarded. The reported heaviness estimates were subject-wise Fischer *z*-transformed. *T*_com_ and the torque planning errors are centered at the person average and thus represented the within person variation (WP) when acting as predictors. The raw digital caliper values indicating the expected and perceived CoMs were centered to the actual grip center of each trial. Similar to Schneider et al. 2019, participants used very different CoM ranges to indicate their expectation and perception of torques. Hence, the caliper CoMs rather reflect individual projections of torques to the caliper range. Therefore, we *Z* standardized the CoM ratings subject wise. As the object CoM and the resulting torques are convertible and are identical after *Z* standardization, we refer to the expected and perceived torque in the following, as torques are the physical dimension to be perceived by grasping. As variables from the previous lift trials were predictors in the statistical model for *T*_com_, we had to discard the observations of the first trial and each trial following observations discarded due to technical errors. Consequently, the datasets contained 2294 observations for the *T*_com_ model and 2358 observations for the perceived torque (*Z*-score) and weight (*Z*-score) models.

#### Statistical analysis

All statistical analyses were conducted in the R environment for statistical computing [version 3.5.0, (R Core Team 2018), R Project for Statistical Computing, Research Resource Identifiers (RRID):SCR_001905] with the packages listed in Schneider et al. 2019. We employed linear mixed-effects regression modelling (LMM) accounting for dependencies due to multiple sampling (Aarts et al. 2014). Random effects are estimates of the variance of the intercepts and slopes of the predictors across subjects. To determine which random effects to include in the model to safeguard against anti-conservative inference (Aarts et al. 2014; Bates et al. 2000; Long 2011), we again adapted the model building approach described by Bates et al. 2015a. In summary, starting with the inclusion of all possible random effects, we iteratively eliminated first the smallest random effects until the model was accommodated by the data. We then iteratively tested whether the remaining random effects significantly contributed to model fit by likelihood-ratio tests between the original, more complex, and the simplified candidate model and only retained the original model if its model fit was significantly superior. After determining the necessary independent random effects, we tested whether including covariances between them improved the model fit.

We fit the LMMs with the restricted maximum likelihood criterion using the ‘lme4’ package [R package: lme4, RRID:SCR_015654, (Bates et al. 2015b)]. Age groups were dummy coded as 0 and 1, such that interactions of the main effects with the age category represent the difference in the main effect between age groups. We will report the findings for both age groups coded as reference group separately. All interactions between the predictors (and predictor interactions) and age group were included as fixed effects (the main effect of age was necessarily also included). The computation of *p* values of fixed effects is based on conditional *F* tests with Kenward–Roger approximation for the degrees of freedom.

Partial regression plots including 95% prediction intervals derived from parametric bootstrapping of the models and the conditional partial residuals graphically represent significant effects. Pseudo *R*^2^ statistics for the fixed effects of the LMMs approximating the portion response variance is explained by the fixed effects are reported as overall measures of goodness of fit. Full model specifications and results can be found in the Supplementary Tables S1, S3, and S4.

We conducted simulation-based post hoc power analyses for prespecified effect sizes to assess the power with which predictors could have been found significant (see Supplementary material, section 2).

##### Modelling *T*_com_

We included the predictors which were found to be significantly correlated with *T*_com_ in the absence of visual handle position cues (Schneider et al. 2019): the external torque of the previous and current trial, and the torque planning error of the previous trial as well as the previously uncorrelated perceived torque of the prior trial. Additionally, the expected torque (*Z*-score) prior to lifting and the external torque induced by the current handle position were selected as fixed effects. The simultaneous inclusion of the total external torque, allowed for the assessment of the impact of the handle position on torque anticipation without a bias by prelift onset corrective actions (Schneider et al. 2019). To evaluate whether the impact of the visual handle cues on torque anticipation changes during subsequent lifting, we included the main effect and the interaction of ‘trials’ with the shape-induced external torque. To test whether the reliance on sensorimotor memories differs between the handle positions, we included the interaction terms between the handle-induced external torque and both the previous external torque as well as the previous planning error (WP). The final model was a random intercept and random slope model including the independent random slopes of the predictors previous and current external torque, external torque induced by the handle position and the expected torque (*Z*-score), i.e. the model estimated the variance of the individual intercepts and mentioned slopes across the individual subjects. Additionally, we fitted models with the same specifications for the torque components *T*_ΔLF*w/2_ and *T*_ΔCoP*GF_ as dependent variables to qualitatively assess differences of the effects on the torque exertion strategy.

##### Modelling the perceived torque (*Z*-score)

Apart from the current external torque and the committed planning error WP which were both highly correlated with the torque percept (*Z*-score) in the absence of visual cues (Schneider et al. 2019), we also included the expected torque and the torque induced by the handle position as main effects. A negative correlation with the object geometry would suggest a predictive subtraction of visually-anticipated torques from the overall percept, i.e. torques were perceived as less strong when they could be anticipated by the object geometry. A positive sign, however, would serve as evidence for an additive integration of visual and sensorimotor influences (Ernst and Bülthoff 2004).

After stepwise evaluation, the random variances of the slopes of the expected torque (*Z*-score), the torque induced by handle position, and the planning error WP as well as the inclusion of all covariances among these were deemed as the appropriate random effect structure for conservative testing of the fixed effects.

##### Modelling the perceived weight (*Z*-score)

We included the trial number as well as the linear and quadratic effect of the external torque, the planning error WP, the handle position, and the expected torque as fixed-effect predictors. The model’s random effect structure consisted of the random slopes of the linear and quadratic effect of the external torque and the linear term of the planning error as well as the covariance between the aforementioned linear terms.

## Results

### Predictive torque control

In the present study the initial peak object tilt was highly correlated with the planning error at lift onset (Pearson’s *R*^2^ = 0.64) confirming the behavioral importance of the torque planning error.

**Fig. 3:**
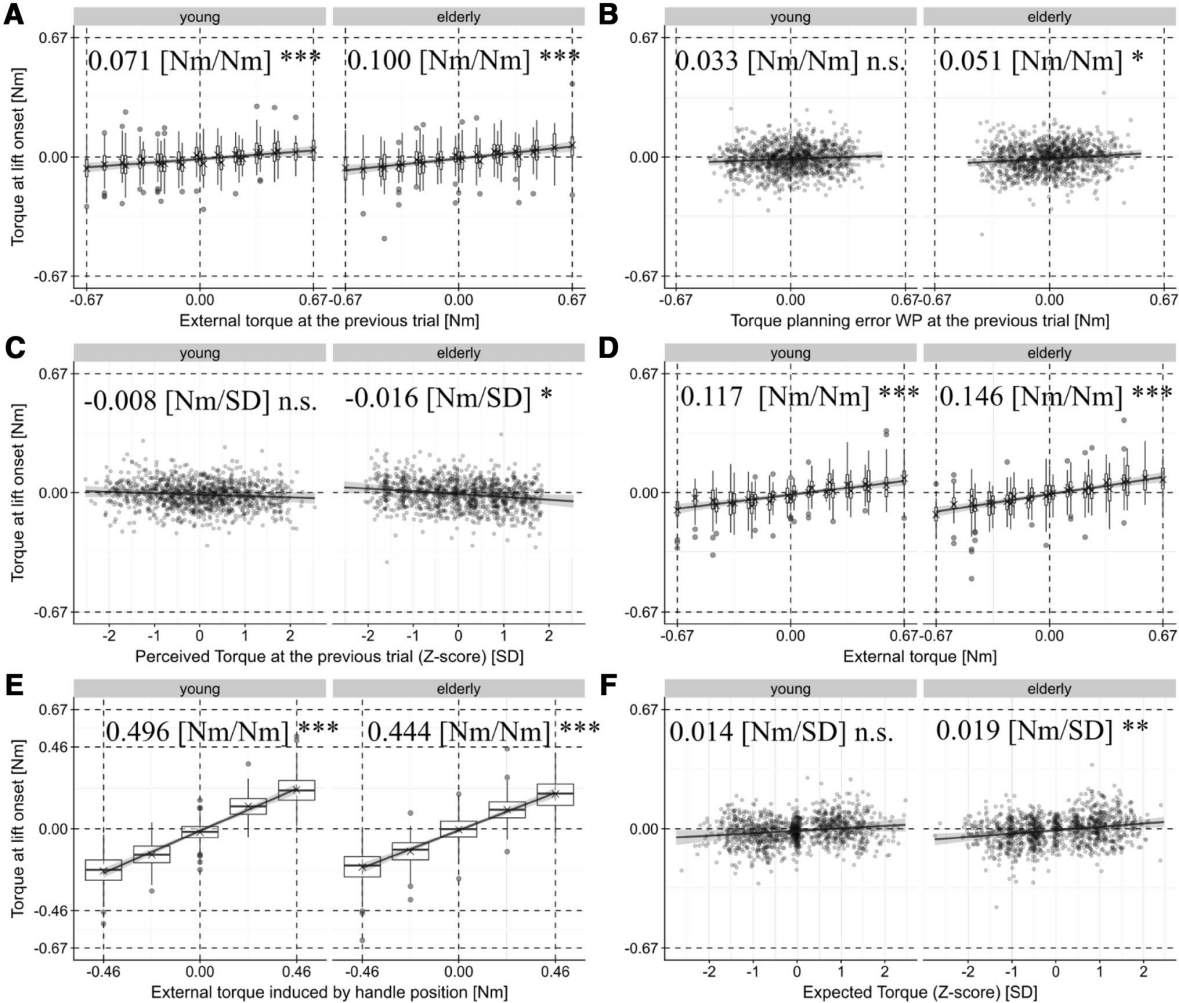
Partial regression plots of the main effects of the linear mixed-effect model for *T*_com_ with 95% confidence intervals of the regression coefficients computed by parametric bootstrapping. The partial residuals are displayed. The effects are separately plotted for both age groups. For predictors with discrete values, we display the distribution of the data with box and whiskers plots in the style of Tukey. The central horizontal line represents the median, while the lower and upper hinges correspond to the 25th and 75th percentiles, respectively. The upper and lower whiskers extend from the upper and lower hinge to the largest value no further than 1.5 * inter-quartile ranges from the respective hinges. Data beyond are plotted individually. Additionally, the means are indicated by ‘*X*’. The regression estimates as well as their significance level are noted

The previous external torque was significantly correlated with *T*_com_ [young 0.071 (Nm/Nm), *t*(295.1) = 3.649, CI 0.033–0.109 (Nm/Nm), *p* =  < 0.001, elderly 0.100 (Nm/Nm), *t*(370.6) = 4.852, CI 0.059–0.140 (Nm/Nm), *p* < 0.001, see Fig. 3a]. We found no significant effect of the previous planning error WP on *T*_com_ for the young and a positive significant correlation for the elderly group [young 0.033 (Nm/Nm), *t*(2209) = 1.534, CI − 0.009 to 0.076 (Nm/Nm), *p* = 0.123, elderly 0.051 (Nm/Nm), *t*(2093) = 2.432, CI 0.010–0.092 (Nm/Nm), *p* = 0.01511, see Fig. 3b]. Thus, we did not replicate the previously found negative correlation between *T*_com_ and the previous torque planning error which signaled a shift of the torque anticipation towards the previous erroneous sensorimotor plan in the absence of cues (Schneider et al. 2019). No significant correlation with the previously-perceived torque was found in the young group, while a negative correlation estimate reached significance in the elderly group [young: − 0.0075 (Nm/SD), *t*(2232) = − 1.180, CI − 0.026 to 0.010 (Nm/SD), *p* = 0.238; elderly: − 0.016 (Nm/SD), *t*(2234) = − 2.381, CI − 0.029 to − 0.003 (Nm/SD), *p* = 0.01734, see Fig. 3c].

Regarding predictors of the current trial, we confirmed an effect of the current external torque on *T*_com_ [young: 0.117 (Nm/Nm), *t*(32.5) = 6.036, CI 0.079–0.155 (Nm/Nm), *p* < 0.001; elderly: 0.146 (Nm/Nm), *t*(32.6) = 7.523, CI 0.108–0.184 (Nm/Nm), *p* < 0.001, see Fig. 3d]. The external torque induced by handle position was correlated with *T*_com_ [young 0.496 (Nm/Nm), *t*(72.8) = 14.40, CI 0.428–0.563 (Nm/Nm), *p* < 0.001, elderly 0.440 (Nm/Nm), *t*(31.4) = 14.147, CI 0.382–0.506 (Nm/Nm), *p* < 0.001, see Fig. 3e]. The regression estimates suggest that almost half of the external torque attributed to object shape was anticipated and incorporated into anticipatory torque compensation. The interaction between the external torque induced by handle position and trial did not reach significance (young: *p* = 0.16, elderly: *p* = 0.23), indicating that the use of geometric cues did not improve in the course of the experiment. We found neither a significant interaction effect between the predictor external torque induced by handle position and the planning error nor between the predictor external torque induced by handle position and previous external torque. Hence, our results do not support the notion of a reciprocal, trial by trial, modulation of the effects of the geometric object shape, and the effects of the planning error and prior external torque on *T*_com_. The correlation of the torque expected prior to lifting (*Z*-score) with *T*_com_ reached significance in the elderly [young 0.0135 (Nm/SD), *t*(38.7) = 1.822, CI − 0.001 to 0.028 (Nm/SD), *p* = 0.076, elderly 0.0187 (Nm/SD), *t*(19.6) = 3.113, CI 0.007–0.030 (Nm/SD), *p* < 0.01, see Fig. 3f]. None of the interactions with age group was statistically significant. The adjusted coefficient of model determination (Pseudo *R*^2^) of the fixed effects indicates that 86.76% of the overall response variance are explained, denoting good model fit. Full model results can be found in the Supplementary Table S1.

Concerning the underlying motor strategy, participants predominantly exerted torques by positioning the fingers at different heights of the grasping surface: 67.4% of the total torque at lift-off was defined by the product of vertical finger distance and grip force (mean ratio $\frac{{T_{{\Delta {\text{COP *GF}}}} }}{{T_{{{\text{com}}}} }}$ = 0.674). The results of separate models fitted with the same specifications for the torque components *T*_ΔLF*w/2_ and *T*_ΔCoP*GF_ as dependent variables, indicate that both the handle positions induced torques as well as previous external torques were more highly correlated with *T*_ΔCOP*GF_ than with *T*_ΔLF*w/2_ (Supplementary Table S2).

### Torque perception (*Z*-score)

**Fig. 4:**
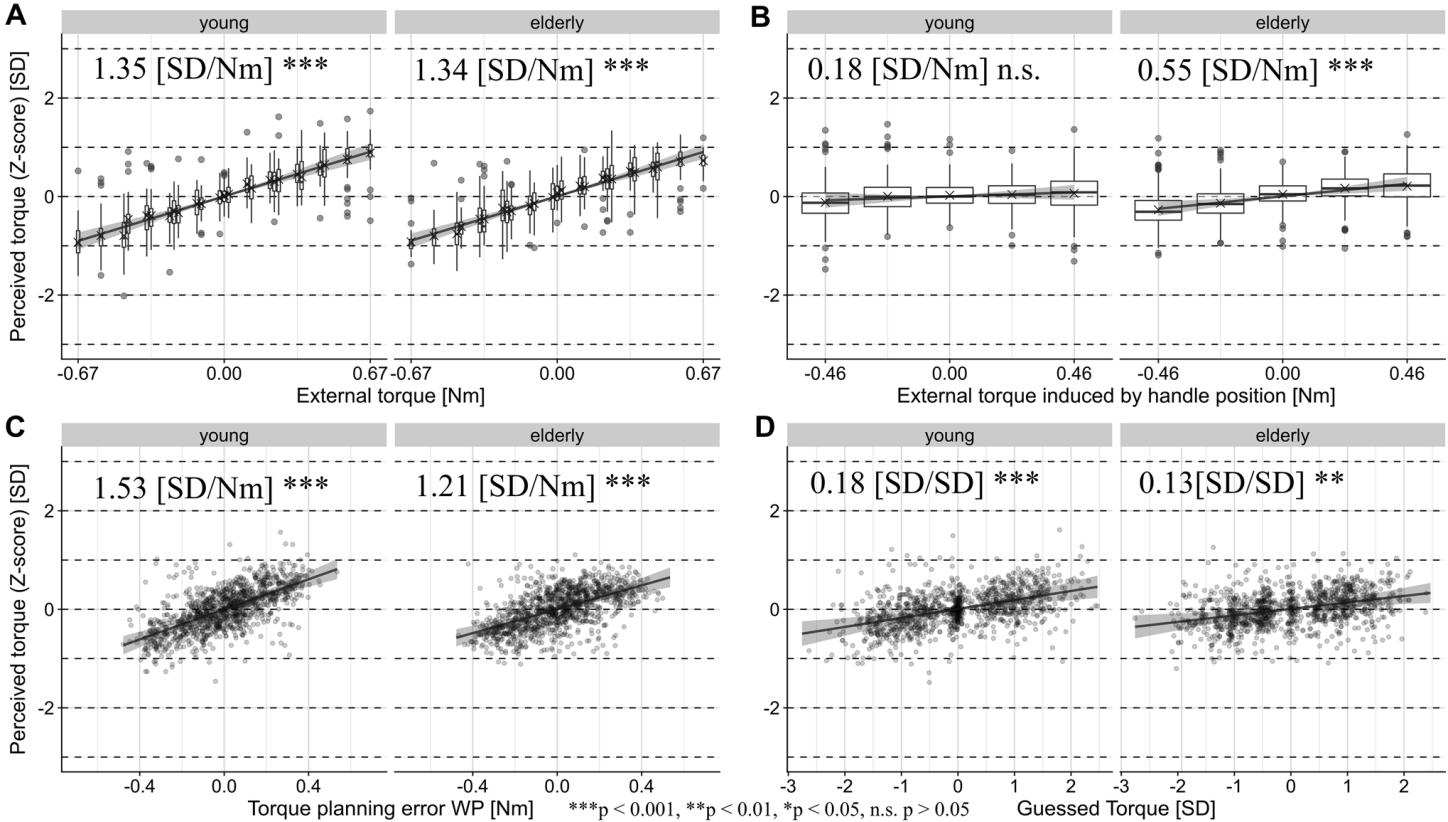
Partial regression plots of the main effects of the linear mixed-effect model for the perceived torque with 95% confidence intervals of the regression coefficients computed by parametric bootstrapping. The partial residuals are displayed. The effects are separately plotted for both age groups. For predictors with discrete values, we display the distribution of the data with box and whiskers plots in the style of Tukey, with additional ‘*X*’ denoting the means. The regression estimates as well as their significance level are noted

Confirming previous findings in the absence of cues (Schneider et al. 2019), the perception of torques was correlated with the actual external torque [young: 1.359 (SD/Nm), *t*(2107) = 10.785, CI 1.112–1.606 (SD/Nm), *p* < 0.001; elderly: 1.336 (SD/Nm), *t*(2092) = 11.649, CI 1.111–1.561 (SD/Nm), *p* < 0.001] and to a similar extent with the committed planning error WP [young: 1.522 (SD/Nm), *t*(42.1) = 7.790, CI 1.139–1.904 (SD/Nm), *p* < 0.001; elderly: 1.216 (SD/Nm), *t*(37.5) = 6.393, CI 0.843–1.588 (SD/Nm), *p* < 0.001]. Given that the range of external torques was 3.2 times higher than in the predecessor study, the effect sizes are comparable to the ones reported previously. The handle position-induced external torque was positively correlated with the perceived torque in the elderly, but did not reach statistical significance in the young [young: 0.163 (SD/Nm), *t*(46.6) = 0.967, CI − 0.167 to 0.492 (SD/Nm), *p* = 0.339; elderly: 0.544 (SD/Nm), *t*(34.6) = 3.591, CI 0.247–0.841 (SD/Nm), *p* < 0.001]. Moreover, the torque perception was also biased towards the initial torque expectations in both groups [young: 0.185 (SD/Nm), *t*(25.5) = 3.906, CI 0.092–0.278 (SD/SD), *p* < 0.001; elderly: 0.136 (SD/Nm), *t*(18.4) = 3.229, CI 0.054–0.219 (SD/SD), *p* = 0.00457]. None of the interactions with age group reached statistical significance. Figure 4 depicts the significant results and the Supplementary Table S3 and the full model specifications and results.

The adjusted coefficient of model determination (Pseudo-*R*^2^) indicates that 87.7% of the total response variance was explained by the fixed effects of the model.

### Heaviness perception (*Z*-score)

The model intercept, i.e. the model prediction of the perceived weight when all predictors are zero, was significantly negative [young: − 0.891 (SD), *t*(2300) = − 16.544, CI − 0.996 to − 0.785 (SD), *p* < 0.001; elderly: − 0.768 (SD), *t*(2297) = − 13.734, CI − 0.878 to − 0.659 (SD), *p* < 0.001], indicating that participants judged the object to feel significantly lighter at the beginning of the experiment with the weight and handle positioned centrally, in the absence of a planning error and when the torque is expected to be zero. Heaviness percepts linearly increased with ongoing trials [young: 0.0086 (SD/trial), *t*(2290) = 10.978, CI 0.007–0.010 (SD), *p* < 0.001; elderly: 0.0060 (SD/trial), *t*(2304) = 7.610, CI 0.004–0.008, *p* < 0.001]. This trial effect was significantly smaller for the elderly participants [interaction between trial and age group elderly: − 0.00262 (SD/trial), *t*(2297) = − 2.366, CI − 0.005 to − 0.000 (SD), *p* = 0.01808].

**Fig. 5:**
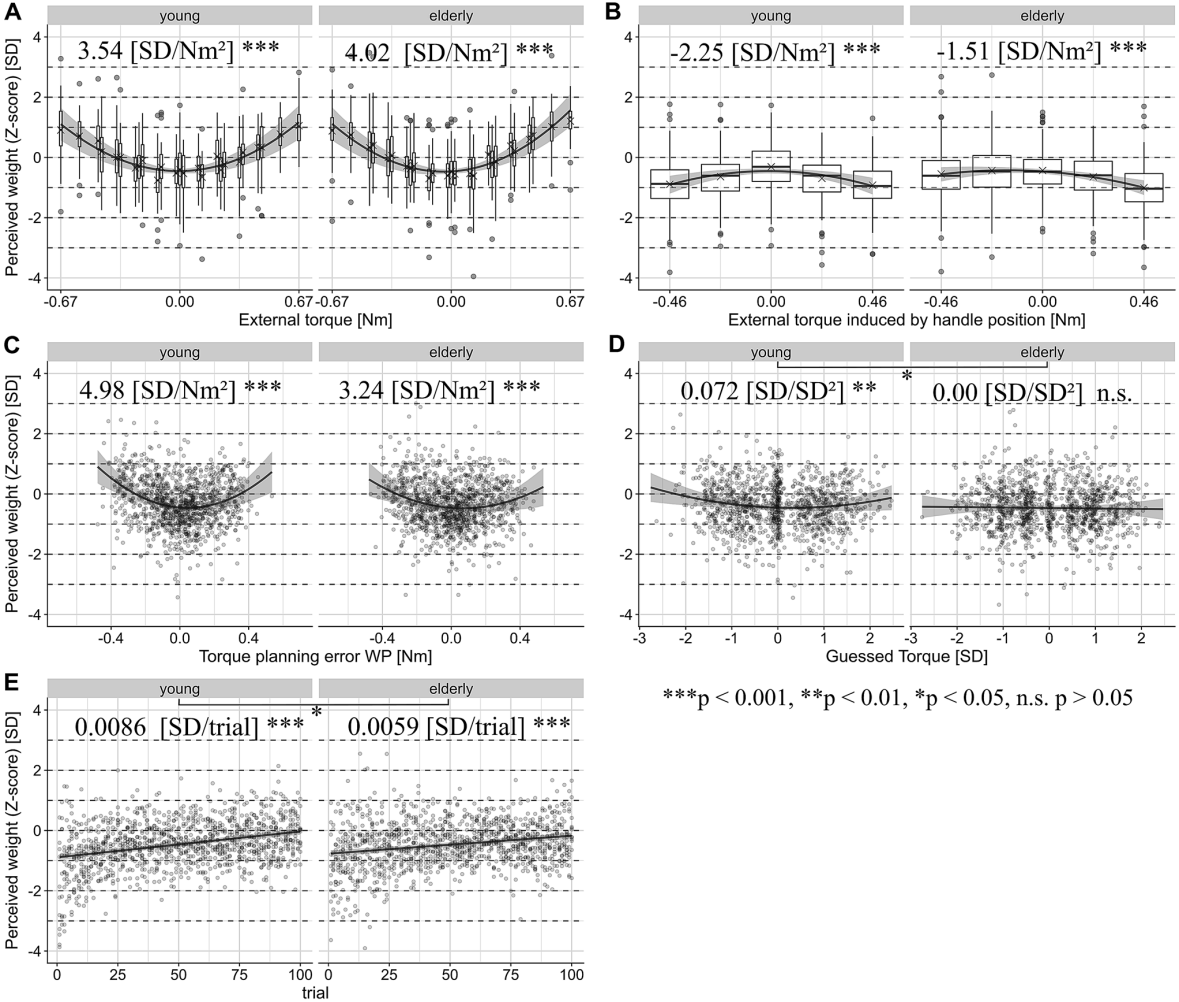
Partial regression plots of the main effects of the linear mixed-effect model for the perceived weight with 95% confidence intervals of the regression coefficients computed by parametric bootstrapping. The partial residuals are displayed. The effects are separately plotted for both age groups. For predictors with discrete values, we display the distribution of the data with box and whiskers plots in the style of Tukey, with additional ‘*X*’ denoting the means. The regression estimates as well as the significance levels of the main effects and significant interactions with age group are noted

Confirming our previous findings in the absence of visual cues, we found a positive quadratic relationship between both the external torque [main effect of external torque^2^: young: 3.541 (SD/Nm^2^), *t*(2305) = 10.191, CI 2.860–4.221 (SD/Nm^2^), *p* < 0.001; elderly: 4.021 (SD/Nm^2^), *t*(2292) = 12.197, CI 3.375–4.668 (SD/Nm^2^), *p* < 0.001] and the planning error WP [main effect of planning error^2^: young: 4.983 (SD/Nm^2^), *t*(111.6) = 5.029, CI 3.041–6.925 (SD/Nm^2^), *p* < 0.001; elderly: 3.243 (SD/Nm^2^), *t*(85.07) = 3.461, CI 1.406–5.079 (SD/Nm^2^), *p* < 0.001]. The linear terms of these predictors were not statistically significant, indicating that the minimum point of the parabola is horizontally centered at zero. The interactions of these predictors with age did not reach significance. The handle position-induced torque was inversely correlated with the perceived heaviness [main effect of external torque induced by handle^2^: young: − 2.251 (SD/Nm^2^), *t*(2301) = − 5.443, CI − 3.061 to − 1.440 (SD/Nm^2^), *p* < 0.001; elderly: − 1.505 (SD/Nm^2^), *t*(2305) = − 3.927, CI −2.255 to − 0.754 (SD/Nm^2^), *p* < 0.001]. Hence, the more eccentric the handle’s position was, the lighter subjects perceived the object to be. The statistically significant, negative linear term in the elderly indicates that the maximum of the parabola was shifted towards a negative torque, e.g. a handle position on the right [elderly: − 0.480 (SD/Nm), *t*(2317) = − 2.111, CI − 0.926 to − 0.034 (SD/Nm), *p* = 0.0349]. In the young group, the expected torque (*Z*-score) was also quadratically correlated with increasing heaviness [main effect of expected torque^2^: young: 0.072 (SD/Nm^2^), *t*(2294) = 2.699, CI 0.020–0.124 (SD/Nm^2^), *p* = 0.00701, elderly: 0.000034 (SD/Nm^2^), *t*(2311) = − 0.001, CI − 0.047 to 0.047 (SD/Nm^2^), *p* = 0.99]. This association was significantly smaller in the elderly [interaction between expected torque^2^ (*Z*-score) and age category elderly: − 0.072 (SD/Nm^2^), *t*(2303) = − 2.003, CI − 0.143 to − 0.002 (SD/Nm^2^), *p* = 0.045]. Figure 5 depicts significant results and Supplementary Table S4 shows full model results.

The adjusted coefficient of model determination (Pseudo *R*^2^) of the fixed effects was 37.1% indicating that only a fraction of the total heaviness percept variance was explained by the model.

## Discussion

Here, participants had to repeatedly grasp and lift an object with a center of mass which was varied trial by trial through alterations of the position of both a visible handle as well as of a hidden weight. Subjects had to prevent object tilt and to indicate their torque expectation prior to grasping as well as their torque and weight perception after having lifted the object. The experiment was designed to allow for evaluation of the contribution of expectations, visual information, haptic information, and recent experience on the anticipatory control of torques as well as the perception of torques and weight in a natural object lifting task by employing mixed-effect multiple regression models.

### Reliance on visual geometric cues for anticipatory torque compensation

We found that the handle position was the most influential factor for anticipatory torque control with participants incorporating just under half of the external torque induced by the handle position into the exerted compensatory torque at lift-off. This stands in line with previous examinations (Fu and Santello 2012, 2015) in which subjects had to grasp constant U- or L-shaped objects at the handle positioned at the edges. In these studies, object geometry switched only between two states and nearly perfect torque anticipation was observed over the course of trials, suggestive of a learning process. Interestingly, the correlation coefficients found here are similar to the initial torque anticipation found in the L-shaped object condition in (Fu and Santello 2012) despite the added random torque variation. Here, the lack of a significant correlation between the handle-induced torque and trial indicates that the utilization of geometric shape cues did not improve with experience suggesting that the experimental design with unforeseen torque variations prevented the formation of direct memory links. Although the previous external torque was again a significant predictor in the present study, the correlation coefficients found here [young: 0.117 (Nm/Nm), elderly: 0.1462 (Nm/Nm)] were obviously smaller than the ones found in the otherwise almost identical predecessor study in which the handle was consistently placed above the center position [young 0.4139 (Nm/Nm), elderly 0.3915, (Schneider et al. 2019)]. Moreover, we did not replicate the negative correlation of the previously committed torque planning errors with *T*_com_ that we interpreted as decreased adaptation towards experienced torques when errors were made in the previous study (Schneider et al. 2019). Instead, we even found a positive correlation between these variables in the elderly. In our opinion, the correlation estimate in the elderly was too small to clearly support the notion that torque planning errors may enhance adaptation towards previous object dynamics. We found no evidence for a trial-to-trial modulation of the weighing of visual and sensorimotor information depending on distinct handle positions or the dynamics of the previous trial as the tested interactions among the handle-induced torque and the previous external torque as well as planning error were both not significant. In agreement with studies showing that neither asymmetric density cues (Craje et al. 2013; Salimi et al. 2003), nor explicit CoM knowledge (Salimi et al. 2000; Zhang et al. 2010) can be utilized for predictive torque compensation, prior torque expectations did not contribute to predictive torque planning in the young. Neither did previous torque percepts, replicating our findings in the absence of cues (Schneider et al. 2019). However, we found these predictors significantly correlated with *T*_com_ in the elderly. As the estimated effect sizes were small, the relevance of these factors for anticipatory torque control remains questionable.

Taken together, our results imply that in grasp to lift tasks, in which relevant geometric cue are available, anticipatory motor planning mainly relies on visual cues whereas the influence of sensorimotor memories of previous task experience is suppressed. Adding random torque variation did not impair the processing of visual geometric cues for torque anticipation, but prevented the formation of direct memory links between shape and torque. These findings stand in line with the findings of Loh et al. 2010 who demonstrated that the influence of sensorimotor weight memories on corticospinal excitability (CSE), which reflect predictive force scaling according to internal models, is suspended when visual weight cues are present. These findings can be reconciled with the present work through the theory of optimal integration. By altering the reliability of visual and haptic cues for size (Ernst and Banks 2002; Van Doorn et al. 2010) and shape judgments (Helbig and Ernst 2007), the CNS was shown to optimally integrate diverging inputs by optimally weighing each source according to its reliability (the inverse of the variance). Optimal integration has also been shown for reaching tasks in which optimal integration of visual and proprioceptive afferences of target location increased pointing accuracy (Beers et al. 1999; van Beers et al. 2002) as well as reaching speed and improved grip aperture control (Camponogara and Volcic 2019). In this experiment, the variance of the torque induced by the handle position around the external torque is much smaller than the variance of the previous external torque around the current external torque, whereas this relation was opposite in studies employing conflicting size cues. Therefore, the suppression of sensorimotor memories in favor of visual cues for torque compensation is the most reasonable behavior which minimizes predictive errors.

Consistent with our previous study (Schneider et al. 2019) but in contrast to (Fu et al. 2010), we found a bias of *T*_com_ towards the current external torque. As the full external torque cannot be inferred until the object is lifted off the surface, we presume that this effect results from a hesitant, probing lifting strategy. On visual inspection of the lift force and object height profiles, we observed that subjects tended to increase their lift forces very slowly prior to surpassing the gravitational force of the object, to possibly lift-off one object edge at a time. This behavior might allow for an at least partial torque feedback and correction of the exerted torque just before the object is completely lifted off. Furthermore, subjects imposed an object lift delay after having matched the gravitational force (of at least one object side) before markably lifting the object. Consequently, *T*_com_ cannot be regarded as an exclusive measure of predictive motor control in this study. Nevertheless, controlling for the effect of the external torque, allowed us to evaluate the predictive influences on *T*_com_.

Concerning the employed motor strategy, the largest part of *T*_com_ was exerted by the product of asymmetric finger partitioning and the grip force *T*_ΔCOP*GF_. Both the handle-induced torques as well as previous external torques were higher correlated with *T*_ΔCOP*GF_ than with *T*_ΔLF*w/2_ (Table S2) in separate models in which the torque components were the dependent variables. This finding stands in contrast with a recent study by Lee-Miller et al. 2016 in which subjects failed to partition their finger positioning according to visual cues. We presume that the differences in the handle geometry and magnitude of the external torques account for this discrepancy as the handle was only half as wide as in the study by Lee-Miller et al. 2016, whereas the maximal external torques were over three time as large. This could have necessitated anticipatory finger positioning to comfortably generate the required torques.

### Haptic sensory input and multimodally-sensed planning errors are the main contributors of torque perception

We replicated our main findings regarding the perception of torques and weight established in the absence of handle position changes (Schneider et al. 2019). Haptic feedback about the external torque and, to the same extent, torque planning errors shaped how participants perceived torques. Again, we propose that sensory information is compared with the sensory predictions based on feed-forward internal models of intended self-generated actions, e.g. the reafference (Bays and Wolpert 2007; Franklin and Wolpert 2011). When sensory predictions and sensory feedback match, the sensory state is perceived weaker than when unpredicted sensory events occur. Here, the tactile feedback of the actual external torque which is probably sensed by integrating fingertip displacement and force patterns during the phase in which the object is held steadily in the air might be subject to sensory attenuation, as has been demonstrated in force-matching tasks (Bays et al. 2006; Shergill et al. 2003). However, Shibata and Santello 2017 reported that the accuracy of the sensing of digit positioning was not affected by active grasping, though. In contrast to the mainly tactile control over the static hold phase (Johansson and Flanagan 2009), tactile, proprioceptive as well as visual afferences, all convey feedback about torque planning errors (i.e. the torque not compensated for at lift-off) and the resulting object tilt. Especially vision might crucially reinforce perception as gaze is predictively directed towards object landmarks during the transition of action phases and aids the judgement of object properties (Flanagan and Johansson 2003; Flanagan et al. 2013). As the errors are unpredicted and occur at a crucial phase of the task, they may be attributed as externally caused and thus be highlighted.

Concerning the impact of geometric cues on torque perception, the handle position-induced torque did not significantly correlate with torque percepts in the young, but did so in the elderly. Contrary to our hypothesis that shape-based feed-forward predictions would be partially subtracted from and hence attenuate the final torque percept, the positive correlation in the elderly age group hints at an additive integration of visual predictions and sensory feedback (Ernst and Bülthoff 2004). Moreover, there was a significant correlation with prior torque expectations in both age groups suggesting that participants tended to confirm their own prior judgments. Judging from the correlation coefficients, the external torque and torque planning error seem to be the principal factors forming the torque percepts. In this experiment, the added random torque variation rendered predictions and expectations based on object shape inferred by vision less reliable than either the tactile and visual sensory experience of mass properties or planning errors gathered during the object lift for the judgement of torques. Although both vision and haptic feedback are affected by noise, visual torque predictions are also affected by the experimentally-induced torque variance. Consequently, from the perspective of optimal multimodal integration, the stronger weighting of sensory input compared to visual predictions and expectations is reasonable.

### Multiple factors modulate the perception of weight

Heaviness ratings increased in the course of trials. Interestingly, this increase was smaller in the elderly which might hint at the elderly being less susceptible or sensitive to physical or, what is more probable, mental fatigue. This finding is consistent with a meta-analysis showing that a preponderance of the literature indicates that older adults develop less muscle fatigue than young adults under a variety of testing conditions (Christie et al. 2011).

We replicated our previous findings that both the experienced torques and the torque planning errors are positively quadratically correlated with heaviness percepts (Schneider et al. 2019). While the increase with torques is probably due to a sense of effort (Flanagan and Bandomir 2000; Flanagan et al. 1995), we reaffirm our claim that cross-modal, sensorimotor error signals, may also induce an illusionary heaviness impression.

As previous studies pointed towards a contrastive comparison of prior-based implicit expectations and sensory feedback (Buckingham 2014), we expected that handle positions at the edges might induce such an expectation of greater torques and hence bigger efforts which might be subtracted from the actual sensory feedback. Indeed, we found a negative quadratic correlation between object shape and heaviness ratings. Concerning the effect of explicit expectations, only the young perceived the object to be significantly heavier when they expected its CoM to lie more eccentrically and this effect was also significantly bigger than in the elderly. In agreement with previous studies on heaviness illusions (Buckingham and Goodale 2013; Buckingham and MacDonald 2016), the effect of these explicit expectations was small.

Taken together, we show that various factors give rise to illusionary heaviness percepts. In addition to the cross-modal sensory impact of external torques and torque errors also found in the absence of geometric cues, we claim that also cross-modal implicit and explicit expectations/predictions based on visual geometric cues can bias our heaviness judgments.

## Conclusion

In summary, we provide evidence that the CNS weighs visual geometric cues and sensory information of external torques and committed planning errors in a task dependent way according to the task specific reliability. While anticipatory torque compensation in a grasp to lift task predominantly relies on geometric cues and suppresses unreliable sensorimotor memories of torques and planning errors, the latter principally shape the way participants perceive torques and object weight. This differential weighing seems a reasonable behavior given the unreliability of previous memories on upcoming torque compensation and the unreliability of visual appearance for judging object properties of hand-held objects. Our principal findings were similar for both age groups, hinting at generalizability across age groups. Nevertheless, we found evidence for slower fatiguability in the elderly and a higher reliance on prior expectations for heaviness perception in the young.

## Electronic supplementary material

Below is the link to the electronic supplementary material.

Supplementary file1 (DOCX 67 kb)
