# Supplementary material for: Visual cues, expectations, and sensorimotor memories in the prediction and perception of object dynamics during manipulation
Source: Exp Brain Res. 2020 Jan 13;238(2):395–409. doi: 10.1007/s00221-019-05711-y (PMC7007906; doi:10.1007/s00221-019-05711-y)
Supplement: Supplementary file 1 — Supplementary file1 (DOCX 67 kb) [file 221_2019_5711_MOESM1_ESM.docx]

Supplementary Material

# Supplementary Tables with full model specifications and results

**Table S1:** Summary of the statistical mixed-effects model of the dependent variable torque at lift onset (T_com_). The fixed effects are represented first with the young and subsequently with the elderly group set as reference group. The residuals variance σ^2^, random effects variances as well as the intraclass correlation coefficients, the number of subjects and observations and the marginal R^2^ (only fixed effects) as well as conditional R^2^ (fixed and random effects) are reported below.

| **Model for T_com_** | | | | |  |  |
| --- | --- | --- | --- | --- | --- | --- |
| **Fixed effects: young group reference** | | | | |  |  |
| *Predictors* | *Estimates* | *CI* | *p* | *df* |  |  |
| Intercept | -0.010 | -0.023 – 0.004 | 0.159 | 55.000 |  |  |
| Trial | -0.000 | -0.000 – 0.000 | 0.733 | 2220.000 |  |  |
| Elderly | 0.008 | -0.011 – 0.027 | 0.434 | 55.000 |  |  |
| External Torque [Nm] | 0.117 | 0.079 – 0.155 | **<0.001** | 33.000 |  |  |
| External Torque induced by handle position [Nm] | 0.496 | 0.428 – 0.563 | **<0.001** | 73.000 |  |  |
| Expected Torque [Z-score] | 0.014 | -0.001 – 0.028 | 0.076 | 39.000 |  |  |
| External Torque 1P [Nm] | 0.071 | 0.033 – 0.109 | **<0.001** | 295.000 |  |  |
| Torque Planning Error 1P WP [Nm] | 0.033 | -0.009 – 0.076 | 0.125 | 2209.000 |  |  |
| Perceived Torque 1P [Z-score] | -0.008 | -0.020 – 0.005 | 0.238 | 2232.000 |  |  |
| External Torque induced by handle position [Nm] x Trial | 0.000 | -0.000 – 0.001 | 0.162 | 2094.000 |  |  |
| External Torque induced by handle position [Nm] x Elderly | -0.052 | -0.143 – 0.039 | 0.269 | 62.000 |  |  |
| Trial x Elderly | -0.000 | -0.000 – 0.000 | 0.435 | 2219.000 |  |  |
| External Torque [Nm] x Elderly | 0.029 | -0.025 – 0.083 | 0.301 | 33.000 |  |  |
| Expected Torque [Z-score] x Elderly | 0.005 | -0.014 – 0.024 | 0.595 | 29.000 |  |  |
| External Torque 1P [Nm] x Elderly | 0.029 | -0.026 – 0.084 | 0.306 | 332.000 |  |  |
| Torque Planning Error 1P WP [Nm] x Elderly | 0.018 | -0.041 – 0.077 | 0.557 | 2176.000 |  |  |
| Perceived Torque 1P [Z-score] x Elderly | -0.008 | -0.026 – 0.010 | 0.371 | 2235.000 |  |  |
| External Torque induced by handle position [Nm] x External Torque 1P [Nm] | 0.028 | -0.037 – 0.093 | 0.397 | 2220.000 |  |  |
| External Torque induced by handle position [Nm] x Torque Planning Error 1P WP [Nm] x | -0.071 | -0.194 – 0.053 | 0.261 | 2215.000 |  |  |
| External Torque induced by handle position [Nm] x Trial x Elderly | -0.000 | -0.001 – 0.001 | 0.865 | 2199.000 |  |  |
| External Torque induced by handle position [Nm] x Elderly x External Torque 1P [Nm] | -0.065 | -0.158 – 0.028 | 0.170 | 2225.000 |  |  |
| External Torque induced by handle position [Nm] x Elderly x Torque Planning Error 1P WP [Nm] | 0.093 | -0.083 – 0.269 | 0.302 | 2217.000 |  |  |
| **Fixed effects: elderly group reference** | | | | |  |  |
| *Predictors* | *Estimates* | *CI* | *p* | *df* |  |  |
| Intercept | -0.002 | -0.016 – 0.011 | 0.756 | 55.000 |  |  |
| Trial | -0.000 | -0.000 – 0.000 | 0.149 | 2218.000 |  |  |
| young | -0.008 | -0.027 – 0.011 | 0.434 | 55.000 |  |  |
| External Torque [Nm] | 0.146 | 0.108 – 0.184 | **<0.001** | 33.000 |  |  |
| External Torque induced by handle position [Nm] | 0.444 | 0.382 – 0.506 | **<0.001** | 52.000 |  |  |
| Expected Torque [Z-score] | 0.019 | 0.007 – 0.030 | **0.006** | 20.000 |  |  |
| External Torque 1P [Nm] | 0.100 | 0.059 – 0.140 | **<0.001** | 370.000 |  |  |
| Torque Planning Error 1P WP [Nm] | 0.051 | 0.010 – 0.092 | **0.015** | 2113.000 |  |  |
| Perceived Torque 1P [Z-score] | -0.016 | -0.029 – -0.003 | **0.017** | 2234.000 |  |  |
| External Torque induced by handle position [Nm] x Trial | 0.000 | -0.000 – 0.001 | 0.234 | 2223.000 |  |  |
| External Torque induced by handle position [Nm] x Young | 0.052 | -0.039 – 0.143 | 0.269 | 62.000 |  |  |
| Trial x Young | 0.000 | -0.000 – 0.000 | 0.435 | 2219.000 |  |  |
| External Torque [Nm] x Young | -0.029 | -0.083 – 0.025 | 0.301 | 33.000 |  |  |
| Expected Torque [Z-score] x Young | -0.005 | -0.024 – 0.014 | 0.595 | 29.000 |  |  |
| External Torque 1P [Nm] x Young | -0.029 | -0.084 – 0.026 | 0.306 | 332.000 |  |  |
| Torque Planning Error 1P WP [Nm] x Young | -0.018 | -0.077 – 0.041 | 0.557 | 2176.000 |  |  |
| Perceived Torque 1P [Z-score] x Young | 0.008 | -0.010 – 0.026 | 0.371 | 2235.000 |  |  |
| External Torque induced by handle position [Nm] x External Torque 1P [Nm] | -0.037 | -0.103 – 0.029 | 0.275 | 2226.000 |  |  |
| External Torque induced by handle position [Nm] x Torque Planning Error 1P WP [Nm] x | 0.022 | -0.104 – 0.148 | 0.731 | 2216.000 |  |  |
| External Torque induced by handle [Nm] x Trial x Young | 0.000 | -0.001 – 0.001 | 0.865 | 2199.000 |  |  |
| External Torque induced by handle position [Nm] x Young x External Torque 1P [Nm] | 0.065 | -0.028 – 0.158 | 0.170 | 2225.000 |  |  |
| External Torque induced by handle position [Nm] x Young x Torque Planning Error 1P WP [Nm] | -0.093 | -0.269 – 0.083 | 0.302 | 2217.000 |  |  |
| **Random Effects** | | | | |  |  |
| σ^2^ | 0.01 | | | |  |  |
| Intercept | 0.00 | | | |  |  |
| External Torque induced by handle position [Nm] | 0.00 | | | |  |  |
| External Torque [Nm] | 0.00 | | | |  |  |
| Expected Torque (Z-Score) | 0.00 | | | |  |  |
| External Torque 1P [Nm] | 0.00 | | | |  |  |
| ICC | 0.05 | | | |  |  |
| N _Subjects_ | 24 | | | |  |  |
| Observations | 2294 | | | |  |  |
| Marginal R^2^ / Conditional R^2^ | 0.884 / 0.890 | | | |  |  |

**Table S2:** Summary of the model estimates and the range of p-values for the final model fitted for T_com_ as well as its components T_ΔLF *w/2_ and T_ΔCoP *GF_ fitted with both age groups as reference group, separately.

|  | model for T_com_ | model for T_ΔLF *w/2_ | model for T_ΔCoP *GF_ |
| --- | --- | --- | --- |
| Handle ind. ext. torque | 0.496^***^/ 0.444^***^ | 0.152^***^/ 0.116^***^ | 0.345^***^/ 0.329^***^ |
| Ext.torque | 0.117^***^/ 0.146^***^ | 0.031^**^/ 0.044^***^ | 0.086^***^/ 0.102^***^ |
| Expected torque (Zscore) | 0.014/ 0.019^**^ | 0.004/ 0.003 | 0.009/ 0.016^**^ |
| Previous ext. torque | 0.071^***^/ 0.100^***^ | 0.025^*^/ 0.014 | 0.044^*^/ 0.085^***^ |
| Previous planning error WP | 0.033/ 0.051^*^ | 0.040^**^/ 0.015 | -0.005/ 0.036 |
| ^***^p < 0.001, ^**^p < 0.01, ^*^p < 0.05 | | | |

**Table S3:** Summary of the statistical mixed-effects model of the perceived torque. The fixed effects are represented first with the young and subsequently with the elderly group set as reference group. The residuals variance σ^2^, random effects variances as well as the intraclass correlation coefficients, the number of subjects and observations and the marginal R^2^ (only fixed effects) as well as conditional R^2^ (fixed and random effects) are reported below.

| **Model for the Perceived Torque** | | | | | |  |  |  |  |  |  |
| --- | --- | --- | --- | --- | --- | --- | --- | --- | --- | --- | --- |
| **Fixed effects: young group reference** | | | | | |  |  |  |  |  |  |
| *Predictors* | *Estimates* | *CI* | *p* | *df* |  |  |  |  |  |  |  |
| Intercept | 0.030 | 0.002 – 0.058 | **0.039** | 2296.000 | |  |  |  |  |  |  |
| Elderly | 0.014 | -0.026 – 0.054 | 0.483 | 2292.000 | |  |  |  |  |  |  |
| External Torque [Nm] | 1.359 | 1.112 – 1.606 | **<0.001** | 2107.000 | |  |  |  |  |  |  |
| External Torque induced by handle position [Nm] | 0.163 | -0.167 – 0.492 | 0.339 | 47.000 | |  |  |  |  |  |  |
| Expected Torque [Z-score] | 0.185 | 0.092 – 0.278 | **0.001** | 25.000 | |  |  |  |  |  |  |
| Torque Planning Error 1P WP [Nm] | 1.522 | 1.139 – 1.904 | **<0.001** | 42.000 | |  |  |  |  |  |  |
| External Torque induced by handle position [Nm] x Elderly | 0.381 | -0.062 – 0.825 | 0.100 | 41.000 | |  |  |  |  |  |  |
| Trial x Elderly | -0.000 | -0.000 – 0.000 | 0.435 | 2219.000 | |  |  |  |  |  |  |
| External Torque [Nm] x Elderly | -0.023 | -0.357 – 0.311 | 0.892 | 2102.000 | |  |  |  |  |  |  |
| Expected Torque [Z-score] x Elderly | -0.049 | -0.173 – 0.075 | 0.450 | 22.000 | |  |  |  |  |  |  |
| Torque Planning Error 1P WP [Nm] x Elderly | -0.306 | -0.840 – 0.228 | 0.268 | 40.000 | |  |  |  |  |  |  |
| External Torque induced by handle position [Nm] x External Torque [Nm] | -0.371 | -0.684 – -0.058 | **0.020** | 2306.000 | |  |  |  |  |  |  |
| External Torque induced by handle position [Nm] x Torque Planning Error WP [Nm] | 0.348 | -0.174 – 0.870 | 0.192 | 2304.000 | |  |  |  |  |  |  |
| External Torque induced by handle position [Nm] x Elderly x External Torque [Nm] | 0.083 | -0.355 – 0.521 | 0.710 | 2302.000 | |  |  |  |  |  |  |
| External Torque induced by handle position [Nm] x Elderly x Torque Planning Error WP [Nm] | -0.626 | -1.359 – 0.108 | 0.095 | 2303.000 | |  |  |  |  |  |  |

| **Fixed effects: elderly group reference** | | | | | | |  | |  | |  | |  | |  | |  |
| --- | --- | --- | --- | --- | --- | --- | --- | --- | --- | --- | --- | --- | --- | --- | --- | --- | --- |
| *Predictors* | *Estimates* | *CI* | *p* | *df* |  | |  | |  | |  | |  | |  | |  |
| Intercept | 0.044 | 0.016 – 0.072 | **0.002** | 2288.000 | | |  | |  | |  | |  | |  | |  |
| Young | -0.014 | -0.054 – 0.026 | 0.483 | 2292.000 | | |  | |  | |  | |  | |  | |  |
| External Torque [Nm] | 1.336 | 1.111 – 1.561 | **<0.001** | 2093.000 | | |  | |  | |  | |  | |  | |  |
| External Torque induced by handle position [Nm] | 0.544 | 0.247 – 0.841 | **0.001** | 35.000 | |  | |  | |  | |  | |  | |  |  |
| Expected Torque [Z-score] | 0.136 | 0.054 – 0.219 | **0.005** | 18.000 | |  | |  | |  | |  | |  | |  |  |
| Torque Planning Error 1P WP [Nm] | 1.216 | 0.843 – 1.588 | **<0.001** | 37.000 | |  | |  | |  | |  | |  | |  |  |
| External Torque induced by handle position [Nm] x Young | -0.381 | -0.825 – 0.062 | 0.100 | 41.000 | |  | |  | |  | |  | |  | |  |  |
| Trial x Elderly | -0.000 | -0.000 – 0.000 | 0.435 | 2219.000 | | |  | |  | |  | |  | |  | |  |
| External Torque [Nm] x Young | 0.023 | -0.311 – 0.357 | 0.892 | 2102.000 | | |  | |  | |  | |  | |  | |  |
| Expected Torque [Z-score] x Young | 0.049 | -0.075 – 0.173 | 0.450 | 22.000 | | |  | |  | |  | |  | |  | |  |
| Torque Planning Error 1P WP [Nm] x Young | 0.306 | -0.228 – 0.840 | 0.268 | 40.000 | | |  | |  | |  | |  | |  | |  |
| External Torque induced by handle position [Nm] x External Torque [Nm] | -0.288 | -0.594 – 0.018 | 0.065 | 2296.000 | | |  | |  | |  | |  | |  | |  |
| External Torque induced by handle position [Nm] x Torque Planning Error WP [Nm] x | -0.278 | -0.793 – 0.237 | 0.291 | 2301.000 | | |  | |  | |  | |  | |  | |  |
| External Torque induced by handle position [Nm] x Elderly x  External Torque [Nm] | -0.083 | -0.521 – 0.355 | 0.710 | 2302.000 | | |  | |  | |  | |  | |  | |  |
| External Torque induced by handle position [Nm] x Elderly x Torque Planning Error WP [Nm] | 0.626 | -0.108 – 1.359 | 0.095 | 2303.000 | | |  | |  | |  | |  | |  | |  |
| **Random Effects** | |  | | | | |  | |  | |  | |  | |  | |  |
| σ^2^ | 0.11 |  | | | | |  | |  | |  | |  | |  | |  |
| External Torque induced by handle [Nm] | 0.16 |  | | | | |  | |  | |  | |  | |  | |  |
| Expected Torque (Z-Score) | 0.02 |  | | | | |  | |  | |  | |  | |  | |  |
| Torque Planning Error [Nm] | 0.28 |  | | | | |  | |  | |  | |  | |  | |  |
| Correlation External Torque induced by handle [Nm] and Expected Torque (Z-Score) | -0.65 |  | | | | |  | |  | |  | |  | |  | |  |
| Correlation External Torque induced by handle [Nm] and Torque Planning Error [Nm] | -0.28 |  | | | | |  | |  | |  | |  | |  | |  |
| Correlation Expected Torque (Z-Score) and Torque Planning Error [Nm] | -0.53 |  | | | | |  | |  | |  | |  | |  | |  |
| ICC | 0.12 |  | | | | |  | |  | |  | |  | |  | |  |
| N _Subjects_ | 24 |  | | | | |  | |  | |  | |  | |  | |  |
| Observations | 2358 |  | | | | |  | |  | |  | |  | |  | |  |
| Marginal R^2^ / Conditional R^2^ | 0.877 / 0.892 |  | | | | |  | |  | |  | |  | |  | |  |

**Table S4:** Summary of the statistical mixed-effects model of the perceived weight. The fixed effects are represented first with the young and subsequently with the elderly group set as reference group. The residuals variance σ^2^, random effects variances as well as the intraclass correlation coefficients, the number of subjects and observations and the marginal R^2^ (only fixed effects) as well as conditional R^2^ (fixed and random effects) are reported below.

| **Model for the Perceived Weight** | | | | |  |  |  |  |
| --- | --- | --- | --- | --- | --- | --- | --- | --- |
| **Fixed effects: young group reference** | | | | |  |  |  |  |
| *Predictors* | *Estimates* | *CI* | *p* | *df* |  |  |  |  |
| Intercept | -0.891 | -0.996 – -0.785 | **<0.001** | 2300.000 |  |  |  |  |
| Trial | 0.009 | 0.007 – 0.010 | **<0.001** | 2290.000 |  |  |  |  |
| Elderly | 0.123 | -0.029 – 0.275 | 0.114 | 2298.000 |  |  |  |  |
| External Torque [Nm] | 0.046 | -0.644 – 0.737 | 0.896 | 138.000 |  |  |  |  |
| External Torque^2^ [Nm^2^] | 3.541 | 2.860 – 4.221 | **<0.001** | 2305.000 |  |  |  |  |
| External Torque induced by handle [Nm] | -0.049 | -0.575 – 0.477 | 0.856 | 2314.000 |  |  |  |  |
| External Torque induced by handle^2^ [Nm^2^] | -2.251 | -3.061 – -1.440 | **<0.001** | 2301.000 |  |  |  |  |
| Expected Torque [Z-score] | -0.049 | -0.575 – 0.477 | 0.856 | 2314.000 |  |  |  |  |
| Expected Torque^2^ [Z-score] | 0.072 | 0.020 – 0.124 | **0.007** | 2294.000 |  |  |  |  |
| Torque Planning Error 1P WP [Nm] | -0.455 | -1.193 – 0.284 | 0.235 | 41.000 |  |  |  |  |
| Torque Planning Error 1P WP^2^ [Nm^2^] | 4.983 | 3.041 – 6.925 | **<0.001** | 112.000 |  |  |  |  |
| Trial x Elderly | -0.003 | -0.005 – -0.000 | **0.018** | 2298.000 |  |  |  |  |
| External Torque [Nm] x Elderly | 0.293 | -0.654 – 1.239 | 0.546 | 122.000 |  |  |  |  |
| External Torque^2^ [Nm^2^] x Elderly | 0.481 | -0.458 – 1.420 | 0.315 | 2300.000 |  |  |  |  |
| External Torque induced by handle position [Nm] x Elderly | -0.431 | -1.121 – 0.258 | 0.220 | 2319.000 |  |  |  |  |
| External Torque induced by handle position ^2^ [Nm^2^] x Elderly | 0.746 | -0.359 – 1.851 | 0.186 | 2303.000 |  |  |  |  |
| Expected Torque [Z-score] x Elderly | 0.029 | -0.102 – 0.160 | 0.667 | 2315.000 |  |  |  |  |
| Expected Torque^2^ [Z-score] x Elderly | -0.072 | -0.143 – -0.002 | **0.045** | 2303.000 |  |  |  |  |
| Torque Planning Error 1P WP [Nm] x Elderly | 0.023 | -1.002 – 1.048 | 0.965 | 38.000 |  |  |  |  |
| Torque Planning Error 1P WP^2^ [Nm^2^] x Elderly | -1.741 | -4.414 – 0.932 | 0.205 | 98.000 |  |  |  |  |
| **Fixed effects: elderly group reference** | | | | |  |  |  |  |
| *Predictors* | *Estimates* | *CI* | *p* | *df* |  |  |  |  |
| Intercept | -0.768 | -0.878 – -0.659 | **<0.001** | 2297.000 |  |  |  |  |
| Trial | 0.006 | 0.004 – 0.008 | **<0.001** | 2304.000 |  |  |  |  |
| Young | -0.123 | -0.275 – 0.029 | 0.114 | 2298.000 |  |  |  |  |
| External Torque [Nm] | 0.339 | -0.309 – 0.986 | 0.308 | 107.000 |  |  |  |  |
| External Torque^2^ [Nm^2^] | 4.021 | 3.375 – 4.668 | **<0.001** | 2292.000 |  |  |  |  |
| External Torque induced by handle position [Nm] | -0.480 | -0.926 – -0.034 | **0.035** | 2317.000 |  |  |  |  |
| External Torque induced by handle position ^2^ [Nm^2^] | -1.505 | -2.255 – -0.754 | **<0.001** | 2305.000 |  |  |  |  |
| Expected Torque [Z-score] | -0.016 | -0.091 – 0.059 | 0.675 | 2318.000 |  |  |  |  |
| Expected Torque^2^ [Z-score] | -0.000 | -0.047 – 0.047 | 0.999 | 2311.000 |  |  |  |  |
| Torque Planning Error 1P WP [Nm] | -0.431 | -1.142 – 0.279 | 0.242 | 34.000 |  |  |  |  |
| Torque Planning Error 1P WP^2^ [Nm^2^] | 3.243 | 1.406 – 5.079 | **0.001** | 85.000 |  |  |  |  |
| Trial x Young | 0.003 | 0.000 – 0.005 | **0.018** | 2298.000 |  |  |  |  |
| External Torque [Nm] x Young | -0.293 | -1.239 – 0.654 | 0.546 | 122.000 |  |  |  |  |
| External Torque^2^ [Nm^2^] x Young | -0.481 | -1.420 – 0.458 | 0.315 | 2300.000 |  |  |  |  |
| External Torque induced by handle position [Nm] x Young | 0.431 | -0.258 – 1.121 | 0.220 | 2319.000 |  |  |  |  |
| External Torque induced by handle position ^2^ [Nm^2^] x Young | -0.746 | -1.851 – 0.359 | 0.186 | 2303.000 |  |  |  |  |
| Expected Torque [Z-score] x Young | -0.029 | -0.160 – 0.102 | 0.667 | 2315.000 |  |  |  |  |
| Expected Torque^2^ [Z-score] x Young | 0.072 | 0.002 – 0.143 | **0.045** | 2303.000 |  |  |  |  |
| Torque Planning Error 1P WP [Nm] x Young | -0.023 | -1.048 – 1.002 | 0.965 | 38.000 |  |  |  |  |
| Torque Planning Error 1P WP^2^ [Nm^2^] x Young | 1.741 | -0.932 – 4.414 | 0.205 | 98.000 |  |  |  |  |
| **Random Effects** | | | | |  |  |  |  |
| σ^2^ | 0.59 | | | |  |  |  |  |
| External Torque [Nm] | 0.42 | | | |  |  |  |  |
| Torque Planning Error [Nm] | 2.69 | | | |  |  |  |  |
| Torque Planning Error^2^ [Nm^2^] | 0.69 | | | |  |  |  |  |
| Correlation External Torque [Nm] and Torque Planning Error [Nm] | -0.64 | | | |  |  |  |  |
| ICC | 0.06 | | | |  |  |  |  |
| N _Subjects_ | 24 | | | |  |  |  |  |
| Corr. | -0.64 | | | |  |  |  |  |
| Observations | 2358 | | | |  |  |  |  |
| Marginal R^2^ / Conditional R^2^ | 0.371 / 0.411 | | | |  |  |  |  |

# Power Analysis

## General considerations

We performed bootstrap simulation-based, post-hoc power analyses using the ‘simr’-package in R(Green and MacLeod 2016). We computed 1000 simulation runs per predefined effect size per investigated predictor. Each run persisted of random data resampling, refitting of the statistical model and obtaining the p value of the predetermined effects by t-tests with Satterthwaite’s method for approximating the degrees of freedom. The indicated power equals the percentage of runs in which the predictors were found significant on a 0.05 alpha level. For each predictor we selected a small, a medium and a large effect size a priori as specified in the subsections. The power for the experimentally observed effect sizes is directly related to the p-values reported in the results-section and was therefore not included in the analyses (Hoenig and Heisey 2001). The power estimates and 95% confidence interval are presented in tables below each subsection. As a convention, a power of 80% is considered sufficient or high and a power over 90% as very high.

## Model for T_com_

Regarding the predictors previous and current external torque, previous planning error and external torque induced by handle position as well as their interactions (except with trial) we suggest that effect sizes of 0.1 Nm/Nm, 0.2 Nm/Nm and 0.3 Nm/Nm are small, medium and large, respectively. Concerning the Z-scored predictors previous torque percept, expected torque and their interactions, we judge an effect to be large when the torque at lift-off changes by 1 SD (~0.233 Nm) per 2 SD. Hence, effect sizes of 0.029125 Nm/SD,0.05825 Nm/SD and 0.1165 Nm/SD were defined as small, medium and large, respectively. The thresholds for the interaction of the handle induced external torque with trial were set to 0.001 1/Nm, 0.002 1/Nm and 0.003 1/Nm.

In summary, the statistical model for T_com_ has a very high power to detect even small effects of the main predictors. Only for a small effect size of the predictor ‘external torque induced by handle position’ we observed only a power of 83.1%. Concerning the interactions of the main predictors with age, only a small interaction effect with the predictor ‘external torque induced by handle position’ could be detected with insufficient power (54,9%). The power to detect small and medium effect sizes of the interaction between the external torque induced by handle position and the previous torque planning error 1P WP [Nm] as well as the respective three-way interaction was insufficient, as was the power to detect small effects of the three-way interaction between the torque induced by handle position, the previous external torque and the age group.

| **Power Analysis: model for T_com_** | | | |  |
| --- | --- | --- | --- | --- |
| *Predictors* | *Small Effect-Size* | *Medium Effect-Size* | *Big Effect-Size* | |
| *Torque predictors and interactions [Nm]* | *0.1 Nm/Nm* | *0.2 Nm/Nm* | *0.3 Nm/Nm* | |
| External Torque [Nm] | 99.90% (99.44, 100.00) | 100.0% (99.63, 100.0) | **100.0% (99.63, 100.0)** | |
| External Torque induced by handle position [Nm] | 83.10% (80.63, 85.37) | 100.0% (99.63, 100.0) | **100.0% (99.63, 100.0)** | |
| External Torque 1P [Nm] | 100.0% (99.63, 100.0) | 100.0% (99.63, 100.0) | **100.0% (99.63, 100.0)** | |
| Torque Planning Error 1P WP [Nm] | 99.80% (99.28, 99.98) | 100.0% (99.63, 100.0) | 100.0% (99.63, 100.0) | |
| External Torque induced by handle position [Nm] x Elderly | 54.90% (51.76, 58.02) | 99.00% (98.17, 99.52) | 100.0% (99.63, 100.0) | |
| External Torque [Nm] x Elderly | 94.80% (93.24, 96.09) | 100.0% (99.63, 100.0) | 100.0% (99.63, 100.0) | |
| External Torque 1P [Nm] x Elderly | 95.00% (93.46, 96.27) | 100.0% (99.63, 100.0) | 100.0% (99.63, 100.0) | |
| Torque Planning Error 1P WP [Nm] x Elderly | 90.60% (88.62, 92.34) | 100.0% (99.63, 100.0) | 100.0% (99.63, 100.0) | |
| External Torque induced by handle position [Nm] x External Torque 1P [Nm] | 83.50% (81.05, 85.75) | 100.0% (99.63, 100.0) | 100.0% (99.63, 100.0) | |
| External Torque induced by handle position [Nm] x Torque Planning Error 1P WP [Nm] x | 36.50% (33.51, 39.57) | 89.00% (86.89, 90.87) | 99.90% (99.44, 100.00) | |
| External Torque induced by handle position [Nm] x Elderly x External Torque 1P [Nm] | 57.60% (54.47, 60.69) | 99.00% (98.17, 99.52) | 100.0% (99.63, 100.0) | |
| External Torque induced by handle position [Nm] x Elderly x Torque Planning Error 1P WP [Nm] | 19.80% (17.37, 22.41) | 61.50% (58.40, 64.53) | 91.30% (89.38, 92.97) | |
| *Perception/ Expectation predictors [SD]* | *0.029125 Nm/SD* | *0.05825 Nm/SD* | *0.1165 Nm/SD* | |
| Expected Torque [Z-score] | 97.20% (95.98, 98.13) | 100.0% (99.63, 100.0) | 100.0% (99.63, 100.0) | |
| Perceived Torque 1P [Z-score] | 99.70% (99.13, 99.94) | 100.0% (99.63, 100.0) | 100.0% (99.63, 100.0) | |
| Expected Torque [Z-score] x Elderly | 85.10% (82.74, 87.25) | 100.0% (99.63, 100.0) | 100.0% (99.63, 100.0) | |
| Perceived Torque 1P [Z-score] x Elderly | 88.60% (86.47, 90.50) | 100.0% (99.63, 100.0) | 100.0% (99.63, 100.0) | |
| *Interactions with Trial [1/Nm]* | *0.001 [1/Nm]* | *0.002 [1/Nm]* | *0.003 [1/Nm]* | |
| External Torque induced by handle position [Nm] x Trial | 97.20% (95.98, 98.13) | 100.0% (99.63, 100.0) | 100.0% (99.63, 100.0) | |
| External Torque induced by handle position [Nm] x Trial x Elderly | 79.30% | 100.0% | 100.0% | |

## Model for the Perceived Torque

For the effect of the torque predictors external torque, torque planning error and external torque induced by handle position and their respective interactions we consider a change of the perceived torque by 2SD for 0.67 Nm (maximum external torque) to be big. Hence, effect sizes of 0.75, 1.5 and 3.0 SD/Nm are considered small, medium and big effect sizes. Concerning the effect of torque expectations on torque perception, we regard effect sizes of 0.25 SD/SD, 0.5 SD/SD and 1.0 SD/SD to be small, medium and big.

In summary, the statistical model for the perceived torque was sufficiently or very highly powered to detect small effects of all main predictors and interactions except the interaction between the torque planning error and the age group (power 77.20%).

| **Power Analysis: model for the perceived torque** | | | |  |
| --- | --- | --- | --- | --- |
| *Predictors* | *Small Effect-Size* | *Medium Effect-Size* | *Big Effect-Size* | |
| *Torque predictors and interactions [Nm]* | *0.75 SD/Nm* | *1.5 SD/Nm* | *3.0 SD/Nm* | |
| External Torque [Nm] | 100.0% (99.63, 100.0) | 100.0% (99.63, 100.0) | **100.0% (99.63, 100.0)** | |
| Torque Planning Error WP [Nm] | 97.30% (96.10, 98.21) | 100.0% (99.63, 100.0) | 100.0% (99.63, 100.0) | |
| External Torque induced by handle position [Nm] | 83.10% (80.63, 85.37) | 100.0% (99.63, 100.0) | **100.0% (99.63, 100.0)** | |
| External Torque induced by handle position [Nm] x External Torque [Nm] | 99.40% (98.70, 99.78) | 100.0% (99.63, 100.0) | 100.0% (99.63, 100.0) | |
| External Torque induced by handle position [Nm] x Torque Planning Error WP [Nm] | 82.70% (80.21, 85.00) | 100.0% (99.63, 100.0) | 100.0% (99.63, 100.0) | |
| External Torque [Nm] x Elderly | 99.50% (98.84, 99.84) | 100.0% (99.63, 100.0) | 100.0% (99.63, 100.0) | |
| Torque Planning Error WP [Nm] x Elderly | 77.20% (74.47, 79.77) | 100.0% (99.63, 100.0) | 100.0% (99.63, 100.0) | |
| External Torque induced by handle position [Nm] x Elderly | 89.50% (87.43, 91.33) | 100.0% (99.63, 100.0) | 100.0% (99.63, 100.0) | |
| External Torque induced by handle position [Nm] x External Torque [Nm] x Elderly | 93.30% (91.57, 94.77) | 100.0% (99.63, 100.0) | 100.0% (99.63, 100.0) | |
| External Torque induced by handle position [Nm] x Torque Planning Error WP [Nm] x Elderly | 93.30% (91.57, 94.77) | 100.0% (99.63, 100.0) | 100.0% (99.63, 100.0) | |
| *Perception/ Expectation predictors [SD]* | *0.25 SD/SD* | *0.5 SD/SD* | *1.0 SD/SD* | |
| Expected Torque [Z-score] | 99.90% (99.44, 100.00) | 100.0% (99.63, 100.0) | 100.0% (99.63, 100.0) | |
| Expected Torque [Z-score] x Elderly | 95.90% (94.48, 97.04) | 100.0% (99.63, 100.0) | 100.0% (99.63, 100.0) | |

## Model for the Perceived Weight

A change of the perceived weight by 2 SD after 100 trials or for a torque of 0.67 Nm or for a torque expectation of 2 SD below or above average represents a big effect. Hence we define small, medium and big effect for the predictor trial as 0.005 /SD, 0.01 /SD, 0.02 /SD, for the squared torque predictors as 0.61s SD/Nm^2^, 1.22 SD/Nm^2^, 2.44 SD/Nm^2^ and for the squared expected torques as 0.25 SD/SD^2^, 0.5 SD/SD^2^ and 1 SD/SD^2^. The same thresholds are set for the interactions with age group.

In summary, the statistical model for the perception of weight was not sufficiently powered to detect small effects with the exception of the predictors trial and expected torque (Z-score) and their interactions. Furthermore, the medium effects of the squared external torque induced by handle position (and the interaction with age group) and even big effects of the squared torque planning error (and the interaction with age group) could only be detected with insufficient power.

| **Power Analysis: model for the Perceived Weight** | | | |  |
| --- | --- | --- | --- | --- |
| *Predictors* | *Small Effect-Size* | *Medium Effect-Size* | *Big Effect-Size* | |
| *Trial and interaction* | *0.005 1/SD* | *0.01 1/SD* | *0.02 1/SD* | |
| Trial [1/SD] | 100.0% (99.63, 100.0) | 100.0% (99.63, 100.0) | 100.0% (99.63, 100.0) | |
| Trial [1/SD] x Elderly | 99.50% (98.84, 99.84) | 100.0% (99.63, 100.0) | 100.0% (99.63, 100.0) | |
| *Squared torque predictors and interactions [Nm]* | *0.61 SD/Nm2* | *1.22 SD/Nm2* | *2.44 SD/Nm^2^* | |
| External Torque^2^ [Nm^2^] | 42.50% (39.41, 45.63) | 93.40% (91.68, 94.86) | 100.0% (99.63, 100.0) | |
| External Torque induced by handle position^2^ [Nm^2^] | 30.20% (27.37, 33.15) | 85.50% (83.16, 87.63) | 100.0% (99.63, 100.0) | |
| Torque Planning Error 1P WP^2^ [Nm^2^] | 9.40% (7.66, 11.38) | 24.70% (22.05, 27.50) | 68.40% (65.42, 71.27) | |
| External Torque^2^ [Nm^2^] x Elderly | 26.30% (23.59, 29.15) | 71.40% (68.49, 74.18) | 100.0% (99.63, 100.0) | |
| External Torque induced by handle position^2^ [Nm^2^] x Elderly | 16.50% (14.25, 18.95) | 58.50% (55.37, 61.57) | 99.30% (98.56, 99.72) | |
| Torque Planning Error 1P WP^2^ [Nm^2^] x Elderly | 6.60% (5.14, 8.32) | 15.40% (13.22, 17.79) | 44.20% (41.09, 47.34) | |
| *Expected torque [SD]* | *0.25 SD/SD* | *0.5 SD/SD* | *1.0 SD/SD* | |
| Expected Torque^2^ [Z-score] | 99.60% (98.98, 99.89) | 100.0% (99.63, 100.0) | 100.0% (99.63, 100.0) | |
| Expected Torque^2^ [Z-score] x Elderly | 92.30% (90.47, 93.88) | 100.0% (99.63, 100.0) | 100.0% (99.63, 100.0) | |

# Literature

**Green P, and MacLeod CJ**. SIMR: an R package for power analysis of generalized linear mixed models by simulation. *Methods in Ecology and Evolution* 7: 493-498, 2016.

**Hoenig JM, and Heisey DM**. The Abuse of Power. *The American Statistician* 55: 19-24, 2001.
